# Bosque County Medical Society Organized

**Published:** 1889-09

**Authors:** 


### KEEP THE BALL IN MOTION.

Dr. P. K. Wortham, of Meridian, a staunch supporter of the JOURNAL, and a co-operator in our endeavor to organize the whole profession of Texas, writes us:

"The Bosque County Medical Society had been dormant for several months, until a short while ago, when we succeeded in getting together a goodly number of M. D.'s, and effected a re-organization, and we are determined now to 'hold the fort.' It is thought in a few more meetings we will enroll in our organization the majority of the physicians of Bosque county.

"In a short time, however, we will lose one of our best and most talented members, Dr. J. H. Wysong, who, being Professor of Chemistry in the Texas Medical College, will remove to Galveston."

